# Convergence of microbial assimilations of soil carbon, nitrogen, phosphorus, and sulfur in terrestrial ecosystems

**DOI:** 10.1038/srep17445

**Published:** 2015-11-27

**Authors:** Xiaofeng Xu, Dafeng Hui, Anthony W. King, Xia Song, Peter E. Thornton, Lihua Zhang

**Affiliations:** 1 Department of Biological Sciences, University of Texas at El Paso, El Paso, TX, 79902, USA; 2 Climate Change Science Institute and Environmental Science Division, Oak Ridge National Laboratory, Oak Ridge, TN 37831, USA; 3 Biology Department, San Diego State University, San Diego, CA 92182, USA; 4 Department of Biological Sciences, Tennessee State University, Nashville, TN 37209, USA; 5 State Key Laboratory of Vegetation and Environmental Change, Institute of Botany, Chinese Academy of Sciences, Beijing 100093, China

## Abstract

How soil microbes assimilate carbon-C, nitrogen-N, phosphorus-P, and sulfur-S is fundamental for understanding nutrient cycling in terrestrial ecosystems. We compiled a global database of C, N, P, and S concentrations in soils and microbes and developed relationships between them by using a power function model. The C:N:P:S was estimated to be 287:17:1:0.8 for soils, and 42:6:1:0.4 for microbes. We found a convergence of the relationships between elements in soils and in soil microbial biomass across C, N, P, and S. The element concentrations in soil microbial biomass follow a homeostatic regulation curve with soil element concentrations across C, N, P and S, implying a unifying mechanism of microbial assimilating soil elements. This correlation explains the well-constrained C:N:P:S stoichiometry with a slightly larger variation in soils than in microbial biomass. Meanwhile, it is estimated that the minimum requirements of soil elements for soil microbes are 0.8 mmol C Kg^−1^ dry soil, 0.1 mmol N Kg^−1^ dry soil, 0.1 mmol P Kg^−1^ dry soil, and 0.1 mmol S Kg^−1^ dry soil, respectively. These findings provide a mathematical explanation of element imbalance in soils and soil microbial biomass, and offer insights for incorporating microbial contribution to nutrient cycling into Earth system models.

Carbon (C), nitrogen (N), phosphorus (P), and sulfur (S) are arguably the four most important elements in global biogeochemical cycling and the C:N:P:S stoichiometry in soils and soil microbes^1^ plays an essential role in biogeochemistry-climate feedback^2^,^3^,^4^. It is well-accepted that all organisms take up these elements from external environments and keep relatively stable concentrations inside their cells to support metabolism^5^,^6^, a phenomenon called stoichiometric homeostasis^7^. This homeostatic regulation is one of the basic properties of organisms, keeping the state of the organisms (e.g., nutrient contents) less variable compared to external supply variations^7^,^8^,^9^.

One applicable example of the homeostasis is the constrained element ratio in living organisms^9^, and the most well-known is the Redfield ratio^10^. Since Redfield reported the well-constrained C:N:P ratio of 106:16:1 in sea water and plankton more than seventy years ago^10^,^11^, many studies have confirmed nutrient stoichiometry as a backbone of ecological theory^6^,^7^,^12^.

Recently, a large number of studies have reported similar Redfield-type ratios in terrestrial ecosystems, particularly for plants^12^ and microbes^1^,^13^. However, there are large variations of this ratio among terrestrial plants and microbes^13^,^14^. It is well-known that soil microbes regulate soil N and P cycling and keep their internal concentrations relatively stable compared to the C:N:P:S stoichiometry in soils^7^. It is unclear, however, how soil microbes regulate internal concentrations of various elements through microbial assimilation. Regarding microbial C, N, P, and S, it is reasonable to expect that the element concentrations in microbial biomass might resemble those in soil organic matter, the primary source of most of these elements. The living organisms, however, may also assimilate individual elements independently, given the various biochemical roles of the different elements^7^.

To explore the microbial assimilation of soil elements, we analyzed a recently compiled global database of elemental concentrations in soils and soil microbial biomass^1^. The objective of this study was to test the hypothesis that there is convergence of microbial assimilation of soil organic carbon across C, N, P, and S; we further evaluated the Redfield-like stoichiometry of microbial biomass and its potential mechanisms and implications. The ratio of elements in soil microbial biomass to those in soil organic matter was used to represent the microbial assimilation of elements, following the similar approach in our previous modeling analysis^15^.

## Results

The newly compiled data of S concentration show that the best-estimates of S concentration are 13.1 mmol S /(Kg dry soil) for soil and 0.3 mmol S/(Kg dry soil) for soil microbial biomass (Fig. S1). Combining these estimates with our previous results for the C:N:P stoichiometry, we estimated the C:N:P:S stoichiometry to be 287:17:1:0.8 for soils, and 42:6:1:0.4 for microbes. We kept P in the stoichiometry as 1 to be comparable with previous estimates^1^.

We tested different regression models including linear, exponential, logarithmic, and power function models and found that a power function can be used to represent the element concentration in microbes and its association with soil element concentrations across various environmental conditions (eqs. 1 & 2):


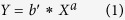



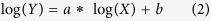


where *X* represents the element concentration in soils; *Y* represents the element concentration in microbes. *a*, *b*′, and *b* are model parameters which might be different for various scenarios of element concentrations, biomes and environmental conditions; *b *= Log(*b*′). It should be noted that the nutrient elements in soil microbial biomass represent only a small portion of those in soils^14^,^16^; we reported soil microbial biomass independently to emphasize the significant roles of microbial biomass^4^,^16^.

**Figure 1:**
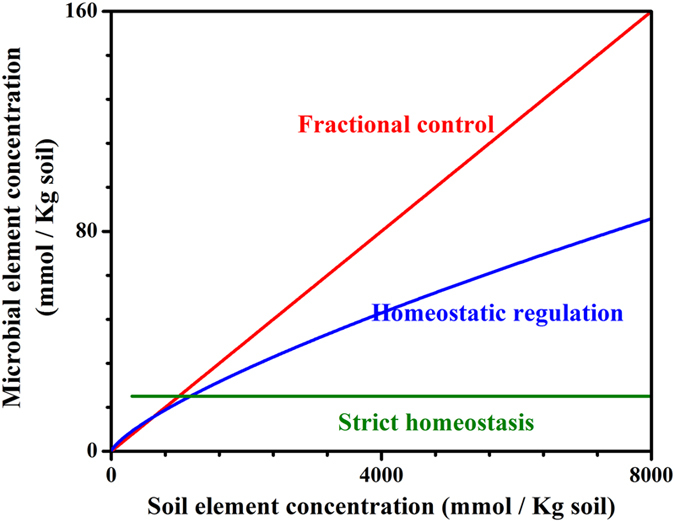
Generic homeostatic regulation of soil elements assimilated by microbes (fractional control represents condition when microbial element is exactly certain fraction of soil elements; homeostatic regulation represents microbial regulation of its element through assimilation; strict homeostasis indicates condition when microbial element concentrations are completely independent of soil element concentration; the three scenarios based on Log(Y) = a × Log(X) + b are, a = 1, b < 0 for fractional control, 0 < *a* < 1, b ≠ 0 for homeostatic regulation, a = 0, b > 0 for strict homeostasis; notice the axis in this figure are not log-transformed which is different from the Eqs. 1 & 2).

We further used a power function to develop the correlation between elements in soils and in soil microbial biomass (methods). Based on the fitted function parameters, we classified the controls of soil element concentrations on microbial element concentrations into three scenarios (Fig. 1): 1) fractional control when *a* *=* *1* and *b* < 0; 2) homeostatic regulation when 0 < *a* < *1* and *b* *≠* *0;* and 3) strict homeostasis when *a* *=* *0*, *b* > *0*. The power function equation has been widely used to describe the homeostatic regulation of nutrients, particularly of N and P in aquatic or terrestrial ecosystems^7^,^13^,^17^,^18^, however, it has not been used to model the single elements in organisms compared to their external environments. For this specific case, the soil microbial element concentration vs. soil elements, *a* is in the range of *(0, 1)*, and *b* in the range of (*−3*, *0*) because 1) microbial element concentration is smaller than soil element concentration, and 2) the difference is less than 3 orders of magnitude. Therefore, the model parameters *a* and *b* could infer the strength in assimilating elements in soil microbial biomass.

**Figure 2:**
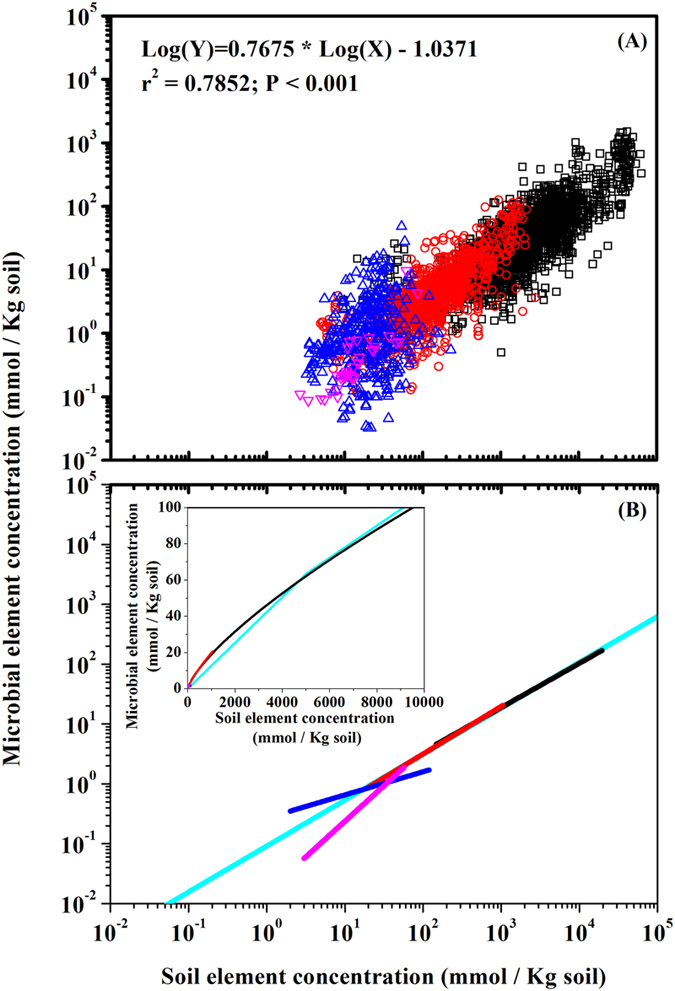
(**A**) Relationship between microbial element concentrations and soil element concentrations (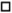 represents carbon (**C**), 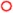 represents nitrogen (N), 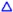 represents P, 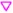 represents S) (**B**) The homeostatic regulation of element concentrations across C, N, P, and S. Inset shows full range of homeostatic regulation (Note: inset has linear x-axis and y-axis. Shallow blue for equation across C, N, P, and S; black is for C, Log(Y) = 0.7391 × Log(X)−0.9407; r^2^ = 0.62; red for N, Log(Y) = 0.7939 × Log(X)−1.087; r^2^ = 0.58; blue for P, Log(Y) = 0.3868 × Log(X)−0.5698; r^2^ = 0.05; and pink for S, Log(Y) = 1.1886 × Log(X)−1.8123; r^2^ = 0.76; all regressions are significant at level of P = 0.05)

The regression line between soil microbial biomass and soil nutrients follows the homeostatic regulation curve for each individual element. Similar regression lines exist for C, N, P, and S, indicating a unifying mechanism for microbial assimilation of soil elements. When we fit all element data together, the overall fitted line is Log*(Y)* *=* *0.7675* (±0.0060) * Log*(X) − 1.0371* (±*0.0174)* with r^2^ = 0.78 and P < 0.001, where *Y* and *X* are element concentrations in soil microbes and soils, respectively (Fig. 2). The slopes of fitted regression lines for each individual element slightly vary among these elements (Fig. 2B; P < 0.1), and the minimum requirement of elements differs significantly (Fig. 2A).

**Figure 3:**
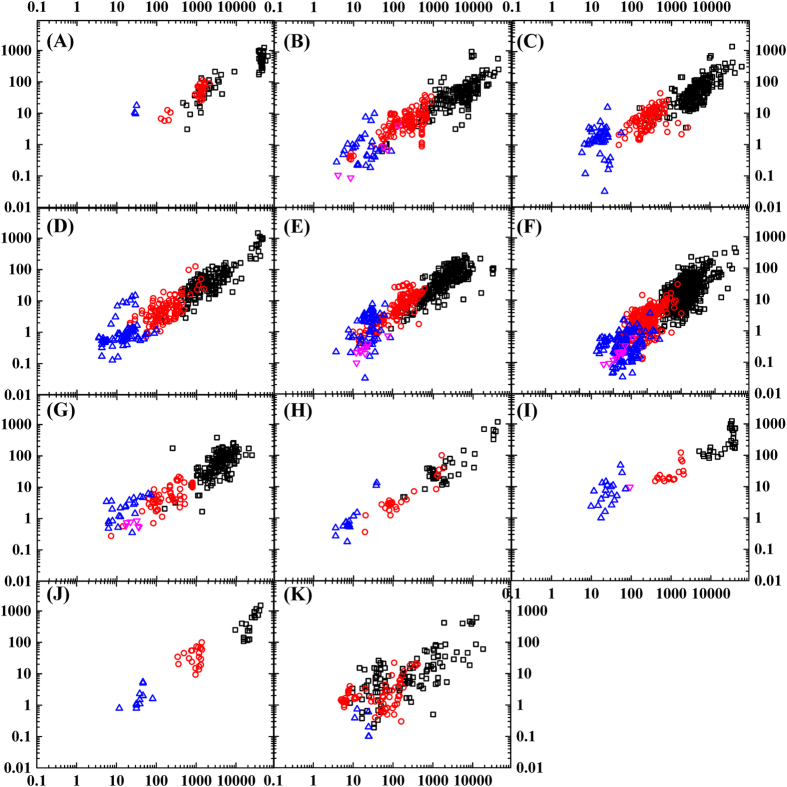
Scatterplot showing C, N, P, and S in soil nutrients and soil microbial biomass for eleven key biomes (S is not included in some biomes due to lack of data; pink reverse solid triangles represent S, blue solid triangles represent P, red solid circles represent N, black solid rectangles represents C; A: boreal forest; B: temperate coniferous forest; C: temperate broadleaf forest; D: tropical/subtropical forest; E: grassland; F: cropland; G: pasture; H: natural wetlands; I: shrub; J: tundra; K: desert/bare soils).

We further partitioned the whole database into eleven different biomes (i.e. boreal forest, temperate coniferous forest, temperate broadleaf forest, tropical/subtropical forest, grassland, cropland, pasture, natural wetland, shrub, tundra, and desert/bare soils) and developed the relationships between microbial element concentrations and soil concentrations for each biome. We found a similar regression of microbial elements in association with soil elements (S was not analyzed in a few biomes due to limited available data here). The biome-level analysis is consistent with our global analysis (Fig. 3 and Table 1). As differences in parameters *a* and *b* across biomes are indicators of differences in homeostatic regulation strength, we compared the values among biomes. Natural wetlands have the lowest *a* value of 0.5713, while the cropland, tundra, and grassland have high *a* values of >0.8, the other biomes have intermediate *a* values. This difference indicates the variations of microbial assimilation of soil elements across biomes; more research is needed to examine the variations and their underlying mechanisms.

Based on the fitted power function (Fig. 2B), we also estimated the threshold of soil element concentration below which there is no detectable soil microbial biomass. By setting the lowest 1% boundaries for microbial C, N, P, and S concentrations in the database, we estimated that minimum requirements of soil elements for soil microbes are 0.8 mmol C Kg^−1^ dry soil, 0.1 mmol N Kg^−1^ dry soil, 0.1 mmol P Kg^−1^ dry soil, 0.1 mmol S Kg^−1^ dry soil for C, N, P, and S, respectively.

## Discussion

The molecular element composition and their concentration in microbes are probably the reason for this convergence among C, N, P, and S. A few previous studies reported element stoichiometry. For example, the protein-to-rRNA ratio could be the origin of Redfield N:P ratio^19^; the ratio of elements in molecular scale could be translated to ecosystem stoichiometry^20^; the ecosystem-level microbial C:N:P:S ratios are caused by the element composition in cells^7^. And recent studies on plant function and stoichiometry confirmed that the allocation to different functions is underlying the elemental composition and stoichiometric shift^21^. Thus the finding in this study indicates that machines in microbial cells as a system are following one unifying mechanism in terms of element assimilation across C, N, P, and S to meet functional demands of various cell machines. More in-depth experiments and analysis to reveal this mechanism are needed.

This finding helps explain the narrower stoichiometry ratios in soil microbial biomass compared to those in soils. Taking concentrations of two elements in soil and microbial element concentrations as an example, we can derive the Eq. 3. Since the parameter *a* is smaller than 1 as shown in this study (Fig. 2A), 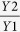 will be narrower than 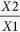, as shown in previous studies on ecological stoichiometry^7^,^13^. Translating to the microbial elements, the C:N:P:S ratios will be narrower than those in the soil elements as shown in many previous studies^13^,^22^.


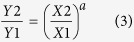


where *Y*_*i*_ is the element concentration in soil microbial biomass, and *X*_*i*_ is the element concentration in soil organic matter; if *Y*_*2*_ and *Y*_*1*_ represent two elements in soil microbial biomass, their corresponding ratio will be *Y*_*2*_*/Y*_*1*_ for microbial biomass and *X*_*2*_*/X*_*1*_ for soil organic matter.

The fitted power function model across C, N, P, and S also explains the well-constrained C:N:P:S stoichiometry in soil microbial biomass. If the element concentrations follow a linear trend, their ratio will be well-constrained, as confirmed by a number of studies^12^,^13^. Meanwhile, the variations would also contribute to the large variation of the C:N:P:S ratios (Fig. 2, Figs S1 and S2). The curve supports the enrichment effect of soil microbes when assimilating elements: soil microbes often hold a relatively high fraction of low-concentration elements in soils, and vice versa^13^,^23^. As Eq. (2) predicts, the element ratio in soil microbial biomass will be narrower than that for soil elements if *a* < 1, which is true for global dataset of C, N, P, and S in soils and soil microbial biomass, as supported by our previous analysis^1^.

The dissimilarities of the homeostatic regulation of microbial element assimilation among biomes could be inferred through comparing the fitted parameters in power function (Table 1). A small *a* value means a relatively narrower ratio in soil microbial biomass, compared to that in the soils, and vice versa. It could be inferred that the cropland, tundra, and grassland have strong potential to enrich low concentration element in microbial biomass while natural wetlands have the weakest potential to enrich low-concentration elements in microbial biomass. This inter-biome discrepancy deserves further investigation.

The model and the scenarios of control described above provide a better understanding of the relationship between elemental concentration in microbial biomass and soils. For example, soil microbial biomass has been expressed as a fraction of total soil nutrient content in some site-level studies^23^,^24^, a case of strict homeostasis (Fig. 1). Others have found that microbes have various enrichment effects for different elements^1^. Normally soil microbes contain a relatively high fraction of soil element if the element is in low concentration in soils (i.e. soil microbial biomass holds 1.2% of soil organic carbon while 8% of total phosphorus). In some cases, there exists an alteration for the element assimilation by soil microbes when the element is highly concentrated, as shown by our homeostatic regulation (Fig. 1)^7^. To sustain microbial biomass, there is a minimum requirement for nutrients; above that threshold of soil nutrient, the microbial assimilation of elements follow homeostatic regulation as proposed in Sterner and Elser (2002)^7^. These minimum thresholds for soil element to sustain microbial biomass have been estimated in the result section.

The reported *a* values in this study are inverse form of *H* (homeostatic regulation coefficient) in Sterner and Elser^7^. Therefore, it is comparable between this research and previous studies regarding the calculated homeostatic regulation^5^,^7^,^25^,^26^. While due to the different organisms and methods used, care should be taken when interpreting the results in this study. For example, Karimi and Folt reported homeostatic regulation for plants^25^, while this study focus on soil microbes. We argue that the unifying mechanisms across C, N, P, and S might be fundamental for understanding microbial control on nutrient cycling in soils and therefore deserves further investigation.

This study reports a unifying mechanism of microbial assimilation of soil elements across C, N, P, and S, based on a recently developed global database of element concentrations in soils and microbial biomass. This study would benefit from a few improvements. From the dataset perspective, the improvements to the data have been identified in our previous publication^1^, same issue prevails and needs to be addressed. For example, apatite P is not directly accessible to microbes although this pool may be ultimately transformed to a microbial accessible form through chemical weathering^27^. Another potential improvement is the soil microbial biomass data; the data used in this study is microbial element concentration on the basis of dry soil; the more accurate data of microbial element on the basis of soil microbial biomass will be more informative and applicable for the analysis. From the methodology perspective, the area-weighted calculation is needed for S in this study. The current estimate of S concentration is not area-weighted due to the lack of data, which might cause biases for C:N:P:S stoichiometry. In addition, the previous studies have reported that biases might be caused by different methods for measuring elements^28^,^29^, and this dataset was compiled with measurements being made with various methods . This bias might not be able to be completely removed, but should be noticed upon interpretation. Last but not least, given the high diversity of soil microbes and the differences of bacteria and fungi for homeostatic regulation^7^, further investigation on different microbial guilds and their contribution to ecosystem level homeostatic regulation is needed.

The implications of this study are multiple-fold. First, the unifying mechanism of microbial assimilations of soil C, N, P, and S indicates that the soil microbes might assimilate elements following a similar path of evolution. Second, the strength of homeostatic regulation of soil elements in soil microbial biomass varies across biomes, indicating the strong environmental and substrate controls on microbial assimilation of soil elements^15^,^30^. Third, the finding of similar trends of microbial assimilation of C, N, P, and S supports the constrained Redfield-like C:N:P:S stoichiometry in soil microbes with soil elements as resources, while the power function concludes a larger variations in terrestrial than marine ecosystems. Fourth, given the importance of microbial control on soil nutrient biogeochemical cycling^27^,^31^ and growing modeling studies incorporating microbial mechanisms into the models^15^,^20^,^32^,^33^, the finding of convergence of microbial assimilation of soil C, N, P, and S in terrestrial ecosystems will provide better solution for simulating of C:N:P:S stoichiometry in plant-microbe-soil system. Fifth, the findings in this study are complementary to ecological stoichiometry theory and element homeostatic regulation in microbial ecology^7^.

## Methods

### Data Compilation

The data on C, N, P, and S in soils and soil microbial biomass were retrieved from published papers. We collected publications by searching for “soil microbial biomass” in Google Scholar and retrieved 3458 data points including 3422 for C, N, and P used in our previous publication^1^, and 36 pairs of S data points for soils and microbes. Associated information for the sampling sites was also retrieved, for example, soil pH, sampling depth, biome type, climate variables, latitude, and longitude. The data points for the top soil layer of 0–30 cm were used in this study. The detailed procedure for data collection and criteria for data screening can be found in Xu *et al*. (2013)^1^. The soil microbial biomass C, N, and P has been archived at Oak Ridge National Laboratory Distributed Active Archive Center for Biogeochemical Dynamics (ORNL-DAAC)^34^, and the soil microbial biomass S data is in the supplementary online material (Table S1).

### Regression Analysis

We first applied log-transformation to all data variables to ensure normal distribution which will be applicable for further statistical analysis. The linear regression on log-transformed data was independently applied to develop correlation between element concentrations in soils and in soil microbial biomass for C, N, P, and S. Then we combined all data for C, N, P, and S and applied linear regression to prove the consistent mechanism of microbial assimilation of C, N, P, and S in terrestrial ecosystems. We further carried out the same statistical analysis for eleven biomes including boreal forest, temperate coniferous forest, temperate broadleaf forest, tropical/subtropical forest, grassland, cropland, natural wetland, pasture, shrubland, tundra, and desert/bare soils. The term bare soils is used to represent a mixed landscape types including bare soils, urban, desert and any other non-vegetated sampling sites. The software Origin Pro 8.0 was used for statistical analysis and generating graphs.

It should be noted that the approach for calculating homeostatic regulation is different from that in Sterner and Elser (2002). First described by French physiologist Claude Bernard in 1865, and coined by Walter Bradford Cannon in1926, the homeostasis infers the ability of living organisms to maintain internal conditions in varying external environments^16^. Sterner and Elser further used a parameter to quantify the homeostatic regulation of living organisms in terms of stoichiometry^7^. In this study, we used the similar method to describe microbial assimilation of soil organic matter across C, N, P, and S. Compared with Sterner and Elser (2002) which depends on the element stoichiometry in living organisms and its external environment, we focus on living organisms regulating its assimilation across elements. Therefore, we use the homeostatic regulation to describe microbial assimilation across C, N, P, and S. This treatment has three reasons: (1) we care more about microbial assimilation mechanisms across elements, rather than individual elements; (2) mathematical function for microbial assimilation across C, N, P, and S informs the variation across elements and for individual elements; (3) the soil microbial biomass accounts for a fraction of soil organic carbon through microbial assimilation of soil organic C, N, P, and S^15^, therefore, the unifying mechanism of microbial assimilation across C, N, P, and S implies consistent elemental ratio in microbes and soils, defined as homeostasis based on Sterner and Elser^7^; while the reverse is not true.

## Additional Information

**How to cite this article**: Xu, X. *et al*. Convergence of microbial assimilations of soil carbon, nitrogen, phosphorus, and sulfur in terrestrial ecosystems. *Sci. Rep*. **5**, 17445; doi: 10.1038/srep17445 (2015).

## Supplementary Material

Supplementary Information

## Figures and Tables

**Table 1:** Model parameters of power function of microbial element concentrations and soil element concentrations for eleven key biomes [values are mean (standard error)] (all regressions are significant at 0.01 level).

Biome	Model parameter		
	*a*	*b*	*r*^*2*^
Boreal Forest	0.6630 (0.0316)	−0.3915 (0.1151)	0.83
Temperate Coniferous Forest	0.7136 (0.0182)	−0.9651 (0.0556)	0.82
Temperate Broadleaf Forest	0.6712 (0.0175)	−0.7493 (0.0552)	0.80
Tropical/Subtropical Forest	0.7617 (0.0165)	−0.9536 (0.0454)	0.85
Grassland	0.8114 (0.0139)	−1.0344 (0.0392)	0.85
Cropland	0.8677 (0.0104)	−1.3846 (0.0290)	0.78
Natural Wetland	0.5713 (0.0303)	−0.1127 (0.1005)	0.85
Pasture	0.7174 (0.0224)	−0.8233 (0.0701)	0.80
Shrubland	0.7565 (0.0465)	−0.9388 (0.1429)	0.86
Tundra	0.8353 (0.0372)	−1.0250 (0.1267)	0.90
Desert/Bare soils	0.6010 (0.0443)	−0.5680 (0.0974)	0.48

*a* is the slope, and *b* is the intercept of the regressed equations for each biome following the equation 1, Log(microbial elements) = a * Log(soil elements) + b; r^2^ is the coefficient of determination of these regressions.
