# Supplementary material for: Convergence of microbial assimilations of soil carbon, nitrogen, phosphorus, and sulfur in terrestrial ecosystems
Source: Sci Rep. 2015 Nov 27;5:17445. doi: 10.1038/srep17445 (PMC4661723; doi:10.1038/srep17445)
Supplement: Supplementary Information [file srep17445-s1.pdf]

Supplementary online material

for

Convergence of microbial assimilations of soil carbon, nitrogen, phosphorus, and sulfur in terrestrial ecosystems

Xiaofeng Xu[1, 2, 3], Dafeng Hui [4], Anthony W. King [2], Xia Song [1, 2], Peter E. Thornton [2], Lihua Zhang [5]

1. Department of Biological Sciences, University of Texas at El Paso, El Paso, TX, 79902, USA; 2. Climate Change Science Institute and Environmental Science Division, Oak Ridge National Laboratory, Oak Ridge, TN 37831, USA; 3. Biology Department, San Diego State University, San Diego, CA 92182, USA; 4. Department of Biological Sciences, Tennessee State University, Nashville, TN 37209, USA; 5. State Key Laboratory of Vegetation and Environmental Change, Institute of Botany, Chinese Academy of Sciences, Beijing 100093, China

Correspondence: X.X. (xxu@mail.sdsu.edu); L.Z. (zhanglihua788403@126.com)

This file contains Figure S1 and S2.

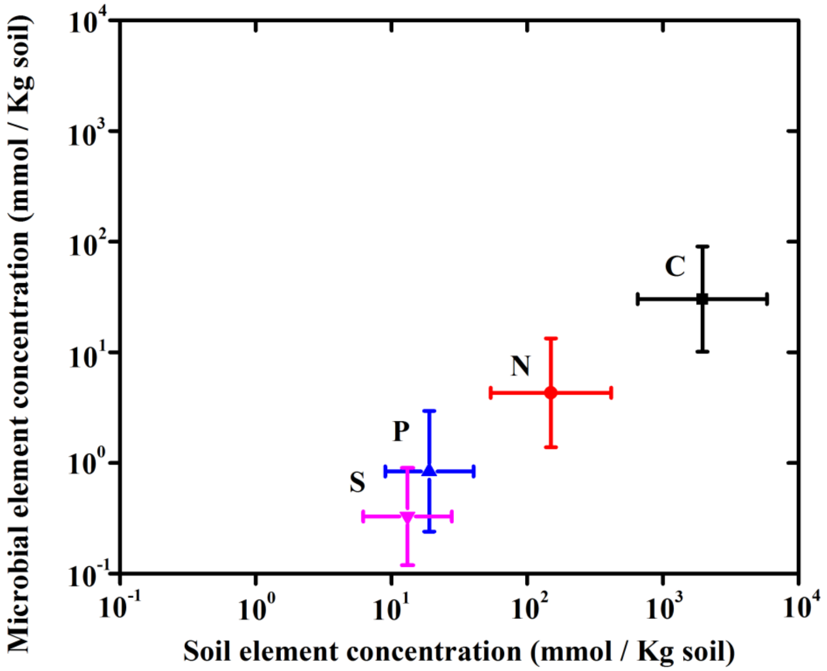

Figure S1 Scatterplot showing globally summarized C, N, P, and S in soil nutrients vs. soil microbial biomass

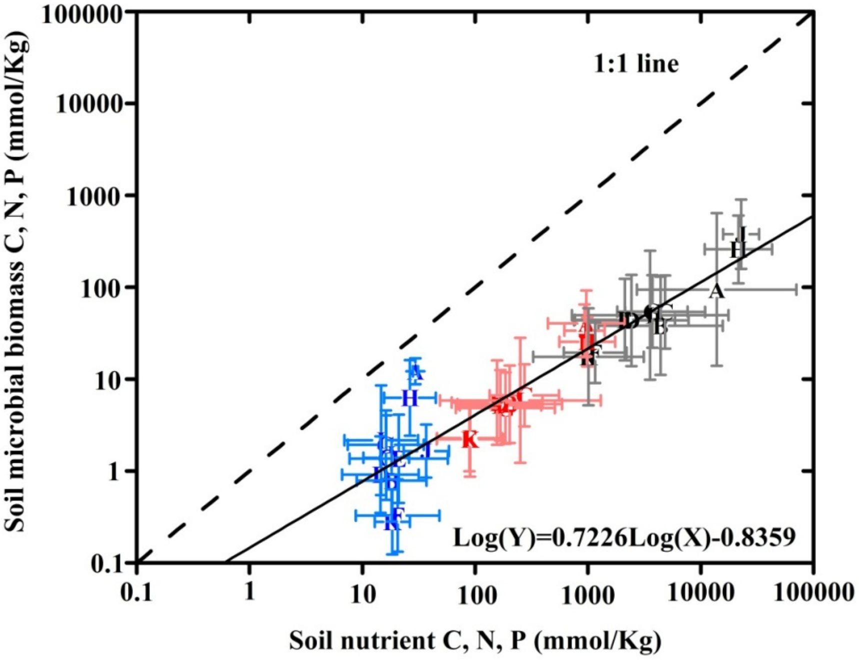

Figure S2. Scatterplot showing average and standard deviation of C, N, and P in soil nutrients and soil microbial biomass at biome level (S is not included in this figure due to lack of data for some biomes; blue triangles represent P, red circles represent N, black rectangles represent C; A: boreal forest; B: temperate coniferous forest; C: temperate broadleaf forest; D: tropical/subtropical forest; E: grassland; F: cropland; G: pasture; H: nature wetlands; I: shrub; J: tundra; K: desert/bare soils)
